# Faith Leaders, Healthy Timing and Spacing of Pregnancies (HTSP), and Family Planning: What Works?

**DOI:** 10.5334/aogh.4072

**Published:** 2023-03-16

**Authors:** Douglas Huber

**Affiliations:** 1 Christian Connections for International Health, Washington, DC, US; 2 Faith to Action Network, Nairobi, Kenya

**Keywords:** healthy timing and spacing of pregnancy, family planning, abortion, contraceptives

We often forget the power of healthy timing and spacing of pregnancies (HTSP) and family planning (FP) services for the health of women and children and to reduce abortions [1,2,3]. Couples who space pregnancies two or more years apart can prevent 10% of child mortality and 30% of maternal mortality [4]. Adequate spacing is also what women want. Data from 27 countries shows 95% of women who are 0–12 months postpartum do not want another pregnancy in the next two years, but only 30% are using effective contraception [5].

In this issue of *Annals of Global Health*, Ochere et al. document the successful engagement of faith leaders in Kenya and Ghana promoting HTSP and the value of family planning. The Channels of Hope approach improved knowledge of HTSP and contraceptive methods HTSP both for women in the study communities and faith leaders in each country [6].

They also recognized that contraceptive use did not change. This signifies that more is required. Many public health initiatives, such as childhood immunization, require not only education and evidence-based information, but also provision of vaccines. Achieving HTSP in practice requires access to quality family planning services and contraceptives. Fortunately, we have good guideposts on how to achieve results.

Faith leaders have shown they can be effective advocates not only for HTSP within their own faith communities, but also for better policies, increased resources, and access to voluntary FP services [7]. In sub-Saharan Africa (SSA) FP uptake can be accelerated through the extensive faith-based networks that own and operate 20–50% of health care facilities in many SSA countries [7,8,9]. Three pillars have provided a solid base for HTSP/FP results: 1) engaging faith leaders to recognize the value of HTSP and voluntary FP and to advocate for the same; 2) training, equipping, and supplying faith-based health facilities; and 3) utilizing community health workers (CHWs) to deliver community-based contraceptives and refer for clinic-based methods. Results included two- to six-fold increases over baseline for a broad range of FP methods, including long-acting reversible contraceptives (LARCs) such as implants [9]. Even in rural Afghanistan two- to four-fold increases in contraceptive prevalence rates were achieved over eight months through community delivery of pills, condoms, and injectables. Mullahs understood the value of adequate spacing and actively educated their communities about HTSP and modern contraceptives during Friday prayers [10].

We can also forget the toll that abortion continues to take in SSA. Unlike other regions abortions have remained high and over three-fourths are unsafe [11]. FP reduces abortion, a benefit appreciated by faith leaders, health providers, and especially women. Where effective contraceptive use increases, unintended pregnancies and abortions drop [12]. It is estimated that USAID global FP efforts in 2019 prevented 7.2 million unintended pregnancies and 3.1 million induced abortions [3].

Updating the knowledge of faith leaders is a first step, given the common misunderstandings about contraceptive side effects and health impact. Faith leaders and faith-based health providers can become trusted advocates and partners with ministries of health (MOH) for training, informational materials, and contraceptives, the latter usually being provided by the MOH. Links with CHWs for delivering community-based methods—pills, condoms, fertility awareness methods, and sometimes injectables—and referrals for the clinic-based reversible methods, such as IUDs and implants, can yield impressive increases in uptake of contraception. [7,9] International faith-based organizations such as World Vision and faith leaders are a good fit for providing evidence-based updates on HTSP and modern contraceptives in SSA, especially when there is follow through with access to voluntary family planning services. They can play a vital role in attaining HTSP, fewer unintended pregnancies and abortions, and better health of women and children.
